# Adrenergic agonist induces rhythmic firing in quiescent cardiac preganglionic neurons in nucleus ambiguous via activation of intrinsic membrane excitability

**DOI:** 10.1152/jn.00761.2018

**Published:** 2019-01-30

**Authors:** Isamu Aiba, Jeffrey L. Noebels

**Affiliations:** Department of Neurology, Baylor College of Medicine, Houston, Texas

**Keywords:** cardiac vagal, epinephrine, nucleus ambiguus, pacemaking, parasympathetic

## Abstract

Cholinergic vagal nerves projecting from neurons in the brain stem nucleus ambiguus (NAm) play a predominant role in cardiac parasympathetic pacemaking control. Central adrenergic signaling modulates the tone of this vagal output; however, the exact excitability mechanisms are not fully understood. We investigated responses of NAm neurons to adrenergic agonists using in vitro mouse brain stem slices. Preganglionic NAm neurons were identified by ChAT-tdTomato fluorescence in young adult transgenic mice, and their cardiac projection was confirmed by retrograde dye tracing. Juxtacellular recordings detected sparse or absent spontaneous action potentials (AP) in NAm neurons. However, bath application of epinephrine or norepinephrine strongly and reversibly activated most NAm neurons regardless of their basal firing rate. Epinephrine was more potent than norepinephrine, and this activation largely depends on α_1_-adrenoceptors. Interestingly, adrenergic activation of NAm neurons does not require an ionotropic synaptic mechanism, because postsynaptic excitatory or inhibitory receptor blockade did not occlude the excitatory effect, and bath-applied adrenergic agonists did not alter excitatory or inhibitory synaptic transmission. Instead, adrenergic agonists significantly elevated intrinsic membrane excitability to facilitate generation of recurrent action potentials. T-type calcium current and hyperpolarization-activated current are involved in this excitation pattern, although not required for spontaneous AP induction by epinephrine. In contrast, pharmacological blockade of persistent sodium current significantly inhibited the adrenergic effects. Our results demonstrate that central adrenergic signaling enhances the intrinsic excitability of NAm neurons and that persistent sodium current is required for this effect. This central balancing mechanism may counteract excessive peripheral cardiac excitation during increased sympathetic tone.

**NEW & NOTEWORTHY** Cardiac preganglionic cholinergic neurons in the nucleus ambiguus (NAm) are responsible for slowing cardiac pacemaking. This study identified that adrenergic agonists can induce rhythmic action potentials in otherwise quiescent cholinergic NAm preganglionic neurons in brain stem slice preparation. The modulatory influence of adrenaline on central parasympathetic outflow may contribute to both physiological and deleterious cardiovascular regulation.

## INTRODUCTION

Central parasympathetic autonomic regulation is predominantly mediated by vagal preganglionic fibers originating in the ventrolateral brain stem medulla oblongata. The caudal portion of the nucleus ambiguus (NAm) contributes the majority of axons in the cardiac vagus nerve and is responsible for cholinergic reduction of heart rate. Chronically reduced vagal tone is implicated in an increased risk of ventricular tachycardia and fibrillation, whereas enhanced tone has antiarrhythmic properties (Kalla et al. 2016). On the other hand, acute vagal hyperactivity may lead to bradycardia, asystoles, atrial fibrillation, vagal reflex syncope, and, in severe cases, cardiac arrest (Alboni et al. 2011; Colman et al. 2004). Strong autonomic coactivation is also arrhythmogenic and evokes complex sympathetic- and parasympathetic-driven cardiac arrhythmias (Koizumi and Kollai 1981), which can be life-threatening in people with heart disease (Shattock and Tipton 2012).

Central adrenergic signaling is likely involved at several levels in the homeostatic response to stress autonomic regulation (Kvetnansky et al. 2009). The descending adrenergic neurons within the rostral ventrolateral medulla brainstem (e.g., A5, C1r groups) project to preganglionic cardiac sympathetic neurons in the intermediolateral cell column of the thoracic spinal cord to modulate cardiac sympathetic outflow (Loewy et al. 1979), and subsets of these adrenergic neurons may also project collaterals to the NAm (Byrum and Guyenet 1987; Kalia et al. 1985; Stocker et al. 1997) to modulate vagal outflow (Abbott et al. 2013). Adrenergic neurons in the locus coeruleus (A6 group) neurons also modulate local inhibitory synaptic transmission in the NAm (Wang et al. 2014). Given the need to maintain tight coordination for optimal sympathetic and parasympathetic balance, central adrenergic feedforward signaling may play important roles in determining cardiac autonomic regulation. However, the mechanisms underlying adrenergic signaling directly onto preganglionic neurons in the NAm are not fully understood. Cardiac vagal neurons do not show pacemaking activity in vitro (Mendelowitz 1996); their activity is considered to be regulated by synaptic activation of postsynaptic ionotropic glutamate, GABA, and glycine signaling, and this input is influenced by various neuromodulators (Dergacheva et al. 2009, 2016; Dyavanapalli et al. 2013; Jameson et al. 2008; Philbin et al. 2010; Sharp et al. 2014). On the other hand, the intrinsic membrane excitability of NAm neurons is enhanced by nonsynaptic neuromodulators, for example, aldosterone depolarization in neonatal rat neurons (Brailoiu et al. 2013b). Currently, the relative importance of synaptic vs. intrinsic membrane excitability mechanisms on the discharge patterns of NAm neurons is not fully understood, nor is it known whether the mechanisms largely studied in immature animals are conserved in the young adult (Kasparov and Paton 1997).

The present in vitro study of young adult NAm cholinergic neurons in mouse brainstem slices shows that adrenergic agonists exert potent nonsynaptic excitatory effects on preganglionic vagal neurons. Although a majority of NAm neurons are quiescent in the absence of synaptic transmission, we found that adrenergic agonists can induce pacemaking activity. This adrenergic excitatory effect produced a robust rhythmic discharge pattern that did not depend on ionotropic synaptic currents, but rather potentiated intrinsic membrane excitability. This mechanism may play a role in coordinate central regulation of sympathetic and parasympathetic autonomic system, and may be a useful target for treatment of abnormal cardiac conditions.

## MATERIAL AND METHODS

### 

#### Animals.

All experiments were conducted under the protocol approved by the Institutional Animal Care and Use Committee at Baylor College of Medicine. ChAT-IRES-Cre mice (stock no. 006410) and *cre*-dependent tdTomato reporter mice (strain Ai9, stock no. 007909) were obtained from Jackson Laboratory and crossed to generate a ChAT-Cre (+/*cre*), tdTomato (+/*flox* or *flox*/*flox*) mouse line. Mice aged between postnatal *days 20* and *50* (P20–P50) of both sexes were used.

#### Retrograde labeling of cardiac premotor neurons in brain stem.

Mice (ChAT-Cre, tdTomato) were deeply anesthetized with avertin (tribromoethanol; 200 mg/kg ip) or 2% isoflurane. Skin overlying the precordial region was depilated and cleansed, and a small vertical skin incision was made along the sternal line. The thoracic wall was exposed, and DiO suspension (30 mg/ml, 100 µl, 30% DMSO in saline) was slowly injected into the pericardial space via the intercostal spaces of the left third to fifth ribs (~1.5-mm depth). Breathing pattern was carefully monitored to ensure absence of pneumothorax. The skin incision was sutured and the mouse allowed to recover for at least 1 wk. For histological analysis, mice were cardiac-perfused with ice-cold PBS followed by 4% paraformaldehyde (PFA). Brain was extracted and kept in 4% PFA overnight at 4°C, followed by further incubation in 30% sucrose until the brain sank. Brain was embedded in OCT compound and cut in 50- to 70-µm coronal sections with a cryostat. Brain sections were rinsed with PBS and mounted on glass slides. Fluorescence images were acquired by fluorescence microscopy (Nikon TE2000S) with the NIS element program and analyzed with ImageJ.

#### In vitro electrophysiology.

Mice were deeply anesthetized with avertin (Tribromoethanol, 200 mg/kg ip), cardiac perfused with a brain cutting solution (in mM: 110 *N*-methyl-d-glucamine, 6 MgSO_4_, 25 NaHCO_3_, 1.25 Na_2_HPO_4_, 0.1 CaCl_2_ 3 KCl, 10 glucose, 0.4 ascorbate, and 1 thiourea, saturated with 95% O_2_-5% CO_2_), and decapitated. The brain was quickly extracted in ice-cold cutting solution, the isolated hindbrain was glued to a mold, the cerebellum was removed, and 200-µm-thick coronal medulla slices were cut with a vibratome (Leica VT-1200). Slices were further hemisected at the midline, incubated in artificial cerebrospinal fluid (ACSF; in mM: 130 NaCl, 3 KCl, 1 MgSO_4,_ 2 CaCl_2_ 25 NaHCO_3_, 1.25 Na_2_HPO_4_, 10 glucose, and 0.4 ascorbate, saturated with 95% O_2_-5% CO_2_) at 32°C for 1 h, and then kept in oxygenated ACSF at room temperature.

Recordings were made in a submerged chamber (RC27; Warner Instruments) continuously perfused with ACSF at 2.5 ml/min and 32–33°C. NAm was visually identified in the transparent regions located in the ventral medulla, and the cholinergic neurons were confirmed by presence of tdTomato fluorescence. All electrophysiological signals were amplified with a Multiclamp 200B amplifier, digitized, and acquired with Clampex software (Molecular Devices). Data were analyzed with pClamp9 (Molecular Devices) and MiniAnalysis software (Synaptosoft).

Cell-attached patch recordings were made by voltage-clamp recording with micropipettes (1–4 MΩ) containing ACSF. Seal resistance was between 5 and 50 MΩ. Data were >1-Hz high-pass filtered. To minimize perturbation of membrane potentials of recorded neurons, the command voltage was adjusted to a voltage at which holding current was within ±300 pA (Perkins 2006). Recordings were included for analysis only when neurons showed spontaneous APs or were silent at rest but with APs that could be evoked by bipolar stimulation (electrodes ~300 µm apart) of adjacent tissue. The maximum AP frequency was calculated from multiple 10-s bins of recorded traces. The peak firing period typically lasts longer than 1 min, and the variability in the duration of induced AP firing does not critically affect the conclusion.

In whole cell recordings, the membrane resistance, capacitance, and access resistance were determined using the membrane test protocol of Clampex software (10-mV, 20-ms rectangular test pulse, at −70-mV holding potential). The measured resistance primarily reflects leak current near the resting potential. In some recordings, data were <100-Hz low-pass filtered. Cells with an access resistance <20 MΩ were accepted for analysis.

Current-clamp recordings were made with potassium gluconate internal solution (in mM: 135 potassium gluconate, 10 HEPES, 1 MgCl_2_, 8 NaCl, 0.05 EGTA, 2 Mg-ATP, and 0.3 Na-GTP, pH adjusted to 7.2 with KOH). Intrinsic membrane excitability was tested after adjustment of bridge balance. Responses to rectangular test pulses (−200 to +450 pA, 50-pA increment, 500 ms) were measured at resting potential or −70 mV to avoid generation of spontaneous APs. Liquid junction potentials were adjusted by 10 mV.

Voltage-clamp recordings were made with a cesium gluconate internal solution (in mM: 130 gluconic acid, 10 HEPES, 1 MgCl_2_, 8 NaCl, 10 BAPTA, 5 TEA, 5 QX-314, 2 Mg-ATP, and 0.3 Na-GTP, pH adjusted to 7.2 with CsOH). Liquid junction potentials were adjusted by 10 mV. Low-voltage-activated currents were evoked by the following current protocol: prepulse at −110 mV for 2 s, followed by voltage step to various potentials (−70 to −30 mV with 10-mV increments). During steady-state inactivation testing (SST), T-type calcium current was evoked with hyperpolarizing prepulses (−110 to −50 mV with 10-mV increments) at 2-s intervals, followed by an activation voltage step to −50 mV. Both activation and SST current-voltage curves were fitted to a sigmoidal curve, and half-maximal activation voltages were calculated. Maximum current amplitude was obtained from the activation curves. Persistent sodium current (*I*_NaP_) was recorded with a cesium gluconate internal solution without sodium channel blocker QX-314. Cadmium (100 µM) was included in the superfusate to reduce voltage-gated calcium currents. Neurons were clamped at −80 mV and slowly depolarized by a voltage ramp (20 mV/s) until +20 mV. After basal current was obtained, recordings were repeated in the presence of tetrodotoxin (TTX; 10 nM, 1 µM) or 30 µM riluzole to isolate the *I*_NaP_ component. Traces averaged from 3–6 sweeps were used for analysis.

#### Drugs.

Epinephrine, norepinephrine, phenylephrine, doxazosin, and isoproterenol were obtained from Sigma Aldrich USA. 1,2,3,4-Tetrahydro-6-nitro-2,3-dioxobenzo[*f*]quinoxaline-7-sulfonamide (NBQX), gabazine (SR95531), carveninolol, and riluzole were obtained from Tocris. TTA-P2 and TTX were purchased from Alomone Laboratories. Riluzole was dissolved in DMSO at 300 mM and stored at −20°C. Other drugs were directly dissolved in ACSF on the day of experiments. The selective T-type calcium channel blocker Z944 was kindly provided by Dr. Terry Snutch (Tringham et al. 2012). GS967 was obtained from MedChemExpress.

#### Experimental design and statistical analysis.

Both male and female mice were used. Because preliminary experiments did not detect a sex difference, data were pooled. All data are means ± SD unless specifically mentioned.

Statistical analyses were conducted using GraphPad Prism and R software. Spontaneous AP frequency change between baseline and exposure to epinephrine (Epi) was tested by Wilcoxon matched-pairs signed-rank test. For repetitive experiments (Figs. 3, 7, and 9), single neurons were exposed to Epi twice, first in control solution and second in the presence of test compound. Quantitative statistical significance in repeated measurements was tested by repeated-measures ANOVA with post hoc Tukey’s multiple comparisons test. Results of ANOVA tests are provided by *F* statistic with *P* values in figure legends. In some pharmacological analyses (Figs. 3, 4, 7, and 8), the fraction of cells that did not respond to Epi in the presence of drug treatment was tested by contingency table analysis with Fisher’s exact test. Cells responding by a >10% increase in their AP frequency were considered to be activated by Epi.

## RESULTS

### 

#### Putative cardiac premotor neurons in the NAm.

**Fig. 1.**
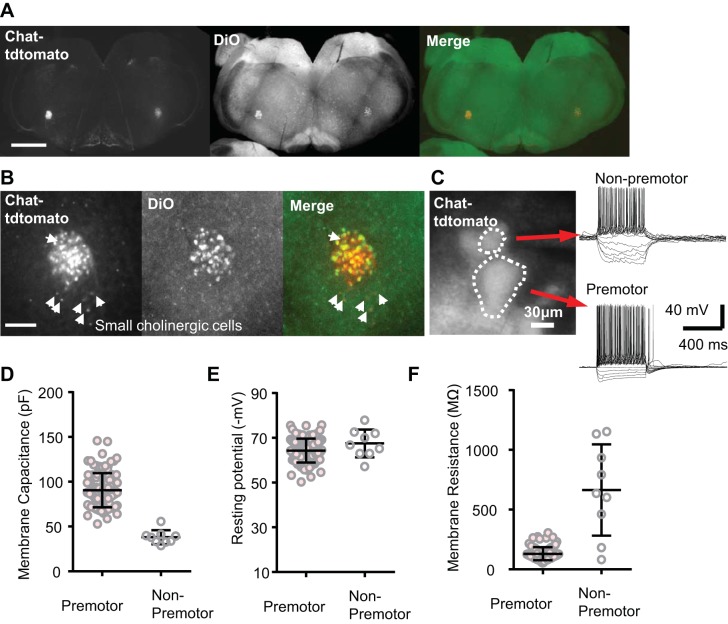
ChAT-tdTomato^+^ labeling of preganglionic neurons within the nucleus ambiguus. *A* and *B*: retrograde tracer DiO was injected into the pericardiac sac, and cells colabeled with retrograde dye and ChAT-tdTomato fluorescence were analyzed. Low magnification (×4, scale bar 1 mm; *A*) and higher magnification (×20, scale bar 400 µm; *B*) images are shown. DiO was detected mostly in cells with a large soma and was absent in the majority of small ChAT-tdTomato^+^ cells (arrowheads), suggesting that most preganglionic cells have a larger soma size. *C*: unlabeled preganglionic and nonpreganglionic ChAT-tdTomato^+^ cells (threshold soma size 40 µm) could be readily distinguished in acute brain stem slices prepared from young adult animals. *D–F*: preganglionic neurons (*n* = 100, soma diameter >40 µm) and nonpreganglionic neurons (*n* = 9) showed distinct membrane excitability. Means and SD are indicated.

Preganglionic neurons in the NAm were identified using a transgenic mouse expressing tdTomato fluorescent protein under the control of the choline acetyl transferase (ChAT) promoter (ChAT-tdTomato). Their cardiac projection was verified by retrograde labeling with a tracer dye (DiO) injected into the pericardial sac (see methods; Fig. 1*A*). All retrogradely labeled DiO^+^ cells were tdTomato positive and had a large soma diameter (>40 µm), whereas ~10% of the ChAT-tdTomato^+^ cells were not labeled with retrograde tracers. These non-cardiac-labeled cells typically had smaller somata and were sparsely distributed within and around the NAm (Fig. 1*B*; *n* = 3 mice). No neurons within the dorsomotor vagal nucleus (DMV) were retrograde-labeled. The lack of DMV labeling may indicate failure to label a subpopulation of cardiac projecting neurons with our method but also indicates little misidentification of gastric preganglionic neurons within NAm (Grkovic et al. 2005).

The electrophysiology of ChAT-tdTomato^+^ cells was characterized in vitro using acute slices prepared from young adult (P20–P50) mouse brain stem. The two populations with different cell sizes could be also distinguished in vitro and showed distinct electrophysiological properties. Consistent with the difference in soma size, these two populations had different mean membrane capacitances (90.9 ± 19.4 vs. 36.6 ± 4.4 pF, *n* = 100, 9; Fig. 1*D*). The resting potentials were similar (−64.5 ± 5.4 vs. −64.1 ± 1.5 mV; Fig. 1*E*). The membrane resistance of neurons with larger soma diameter was low and relatively homogenous, whereas membrane resistances of the smaller neurons differed across a larger range (130.0 ± 53.8 vs. 663.4 ± 382.0 MΩ; Fig. 1*F*). These results suggest that the ChAT-tdTomato^+^ cells with a small soma are a mixed population and may be cholinergic local circuit or oropharynx/larynx projection neurons (Irnaten et al. 2001). Based on histology and electrophysiological characterizations, the ChAT-tdTomato^+^ cells with larger soma (>40-µm diameter) are presumed to be the long-range cardiac preganglionic neurons, and in subsequent experiments, all analyses were made from this cholinergic neuron group.

#### Adrenergic agonists activate spontaneous rhythmic AP firing in NAm neurons in part via α_1_- and β-receptor mechanisms.

**Fig. 2.**
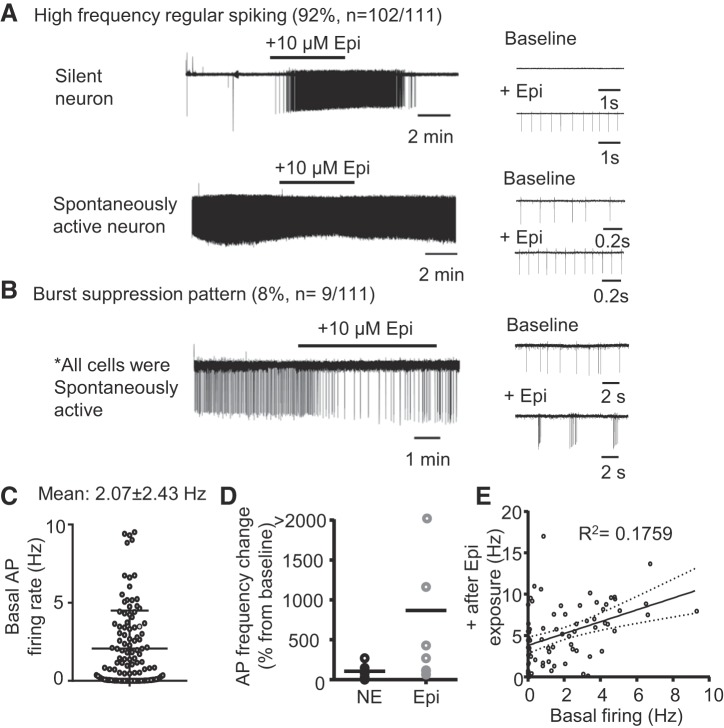
Adrenergic agonist activation of nucleus ambiguus (NAm) neurons in vitro. *A*: bath application of epinephrine (Epi; 10 µM) reversibly increased spontaneous action potential (AP) rate in silent (*top*) and spontaneously active (*bottom*) neurons. *B*: in some NAm neurons (8%, 9/111 cells), Epi modulated NAm firing in a burst suppression pattern. All of the burst neurons were spontaneously active. *C*: summary data of basal spontaneous AP rate of recorded neurons (*n* = 111). Mean and SD are indicated. *D*: comparison of AP frequency changes by repetitive Epi and norepinephrine (NE) exposures. NE had a less potent excitatory effect [*n* = 7 neurons, repeated-measures ANOVA: *F*(1, 12) = 19.62, *P* = 0.0008]. Means are indicated. *E*: a linear regression analysis of AP firing rate at baseline and after Epi exposure. A correlation was detected (*R*^2^ = 0.1759, *P* = 0.0002). Solid line is regression line; dashed lines indicate 95% confidential interval.

Adrenergic signaling could regulate NAm cholinergic output through modulation of excitatory and inhibitory synaptic transmission, as shown by subtype-selective adrenoceptor agonist/antagonist in vitro studies (Bateman et al. 2012; Boychuk et al. 2011; Philbin et al. 2010; Sharp et al. 2014). However, the possibility of direct postsynaptic effects has not yet been clearly demonstrated. We recorded NAm AP firing using cell-attached microelectrode recordings. Under basal conditions, NAm cells showed a variety of spontaneous AP firing patterns in vitro, ranging from complete silence to active firing (2.07 ± 2.43 Hz, *n* = 111 neurons; Fig. 2*C*). Bath application of Epi (10 µM) or norepinephrine (NE; 10 µM) significantly enhanced spontaneous AP firing in NAm neurons (Fig. 2*A*). Epinephrine increased the spontaneous action potential rate in a majority of these cells (92%; 102/111 cells), whereas the remainder (8%; 9/111 cells) displayed a “burst suppression” firing pattern (Fig. 2*B*). Because of the low chance of encountering the burst cells, further characterization focused on the regular-spiking cells. Linear regression analysis detected a positive correlation between firing rate in baseline and after Epi exposures across the regular-spiking neurons (*P* = 0.0002; Fig. 2*E*).

Epi had a stronger excitatory effect than NE. Comparison of efficacy was made by sequentially exposing single neurons with Epi and NE (4 neurons were first exposed to Epi washout and then to NE; the other 3 neurons were exposed to NE, washed, and then exposed to Epi). Epi increased spontaneous AP rate 9.7 ± 1.4-fold, whereas NE increased AP rate 2.1 ± 0.9-fold in the same neurons (*n* = 7; Fig. 2*D*). Because of its higher efficacy, the majority of subsequent experiments were performed using Epi as an agonist.

We next examined the adrenoceptors involved in adrenergic activation of NAm neurons. To ensure regular-spiking response to Epi, NAm neurons were first exposed to Epi, washed, and then reexposed to Epi with or without a selective antagonist for each test compound.

**Fig. 3.**
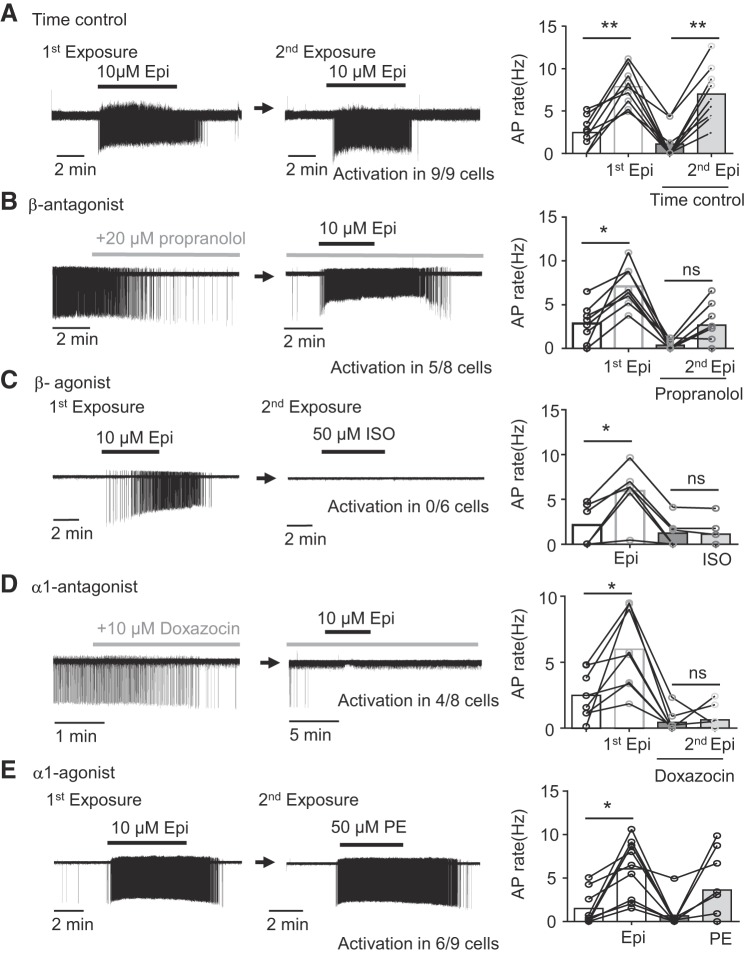
α_1_- and β-adrenergic receptors differentially contribute to nucleus ambiguus (NAm) excitability. *A–E*: NAm responses to adrenergic agonist and antagonists. Single NAm neurons were exposed to epinephrine (Epi) twice, first in control condition and second in the presence of test compound. The action potential (AP) rate (Hz) indicates the maximum instantaneous AP frequency calculated from multiple 10-s bins. Representative traces and quantitative analysis are shown. *A*: a time control experiment. Epi exposure (10 µM; 3–5 min; horizontal bars) reproducibly activated NAm neurons without rundown of peak firing frequency [repeated-measures ANOVA, *F*(3, 8) = 29.63, *P* < 0.0001]. *B*: bath application of β-blocker propranolol reduced basal firing rate and attenuated adrenergic activation [occluded in 3/8 cells; repeated-measures ANOVA, *F*(3, 7) = 22.39, *P* < 0.0001]. Traces show response to propranolol (*left*) and same cell exposed to Epi in the presence of propranolol (*right*). *C*: in contrast, β-agonist isoproterenol (Iso; 10–50 µM) failed to induce AP in all cells tested [activation in 0/6 cells; repeated-measures ANOVA, *F*(3, 5) = 20.64, *P* = 0.0010]. *D*: α_1_-blocker (doxazosin) also reduced spontaneous AP rate and attenuated or prevented NAm activation by Epi in 4/8 cells [repeated-measures ANOVA, *F*(3, 7) = 19.46, *P* = 0.0002]. Traces show response to doxazosin (*left*) and the same cell exposed to Epi in the presence of doxazosin (*right*). *E*: unlike β-agonist, α_1_-agonist phenylephrine (PE) alone could activate 66% (6/9 cells) of NAm neurons [repeated-measures ANOVA, *F*(3, 8) = 9.002, *P* = 0.0024]. *P < 0.05; **P < 0.01; ns, not significant.

In time-controlled experiments, first and second Epi exposures increased spontaneous AP rate of the NAm neurons to a similar degree (*n* = 9; Fig. 3*A*). In experiments with the pan-β-receptor antagonist propranolol (10 µM), wash-in of the antagonist reduced the basal spontaneous AP firing rate (76.3 ± 27.8% reduction). The antagonist also blocked AP increases in 38% (3/8 cells) of cells tested and reduced the increase of AP frequency in the rest of cells (Fig. 3*B*). In contrast, bath application of the β-receptor agonist isoproterenol (10–50 µM) did not increase AP firing in all cells tested (*n* = 6; Fig. 3*C*). These results suggest a partial inhibitory effect of β-receptor antagonist. The lack of an agonist effect could indicate saturation of β-receptor activity in the basal condition, although a nonspecific action on other sites is also possible. Similar to the β-blocker, the α_1_-antagonist doxazosin also reduced basal AP rate by 92.8 ± 11.8% and prevented AP induction in 50% (4/8 cells) of neurons (Fig. 3*C*). In contrast to the β-agonist, the α_1_-agonist (10–50 µM) phenylephrine (PE) partially mimicked the Epi effect and increased spontaneous AP discharge in 66% (6/9 cells) of NA cells (Fig. 3*D*), whereas it was without effect in the remainder.

These results collectively indicate the predominant roles of α_1_-receptors in epinephrine-dependent NAm activation pathways; the β-pathway may contribute to the maintenance of basal excitability but is unlikely to contribute to AP induction.

#### AP induction by adrenergic agonists can occur independently of synaptic input.

Subtype-selective adrenergic agonists/antagonists have been shown to modulate synaptic transmission in NAm cardiac vagal neurons recorded in neonatal rat brain stem slices (Bateman et al. 2012; Boychuk et al. 2011; Philbin et al. 2010; Sharp et al. 2014; Wang et al. 2014). We examined whether such synaptic modulation fully explains the adrenergic activation seen in NAm neurons studied in the young adult mouse.

**Fig. 4.**
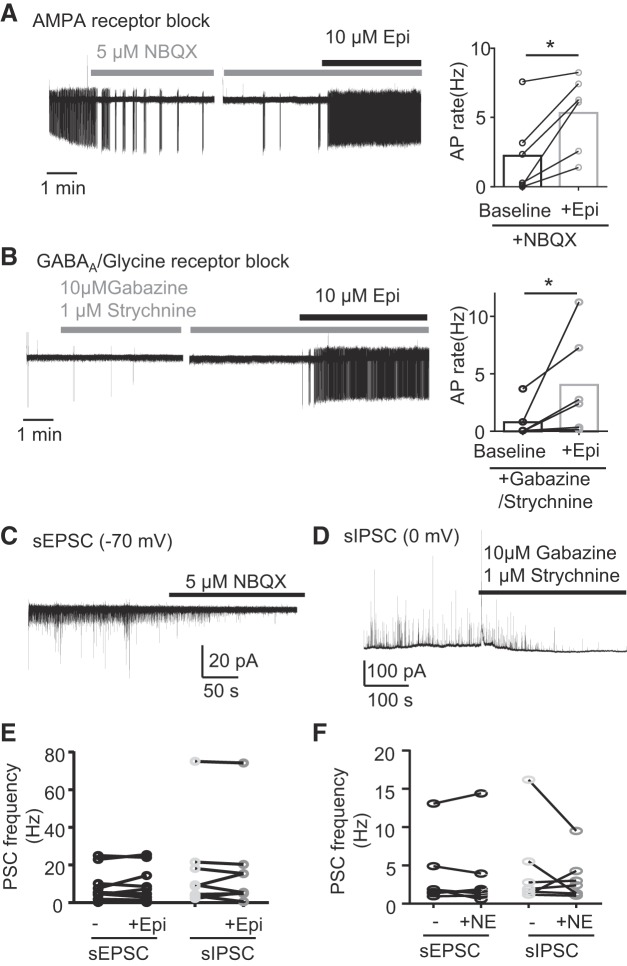
Adrenergic activation does not require synaptic mechanism. *A*: inhibition of postsynaptic AMPA/kainic acid receptors by 1,2,3,4-tetrahydro-6-nitro-2,3-dioxobenzo[*f*]quinoxaline-7-sulfonamide (NBQX) significantly reduced basal firing rate of nucleus ambiguus (NAm) neurons but did not prevent activation by epinephrine (Epi; AP rate increase in 5/6 cells; *P* = 0.0313, Wilcoxon matched-pairs signed rank test). Traces show response to NBQX (*left*) and same cell exposed to Epi in the presence of NBQX (*right*). Quantitative analysis is shown in bar graph. *B*: similarly, inhibition of inhibitory synaptic transmission by gabazine and strychnine did not prevent the adrenergic activation of NAm neurons (AP rate increase in 6/6 cells; *P* = 0.0313, Wilcoxon matched-pairs signed rank test). Traces show response to gabazine and strychnine (*left*) and same cell exposed to Epi in the presence of gabazine and strychnine (*right*). Quantitative analysis is shown in bar graph. *C* and *D*: NBQX and gabazine-strychnine cocktail used in these experiments effectively inhibited spontaneous excitatory (sEPSC) and inhibitory postsynaptic currents (sIPSC), respectively. *E* and *F*: Epi or norepinephrine (NE), respectively, did not significantly modify sEPSC or sIPSC frequency in NAm neurons under the condition where spontaneous AP was reliably induced. *P < 0.05.

In a control experiment, direct bath application of 300 µM glutamate reversibly increased AP frequency of NAm neurons (*n* = 2; in agreement with previous studies: Brailoiu et al. 2013a, 2013c, 2014; Sampaio et al. 2014; Yan et al. 2009), supporting the possible contributing role of excitatory synaptic transmission in adrenergic activation of NAm neurons. However, this is unlikely to provide the sole explanation. Although bath application of NBQX (10 µM), the AMPA/kainate receptor antagonist, significantly decreased predrug AP discharge rate in spontaneously active neurons (−58.8 ± 40.4% change from predrug baseline frequency, *n* = 6; Fig. 4*A*), Epi still increased the AP firing rate in 83% (5/6 cells) of the NAm neurons (Fig. 4*A*). These results indicate that tonic glutamate receptor activation supports the spontaneous activity of NAm neurons but is not required for AP induction by adrenergic agonists.

We also tested whether inhibitory control was similarly affected, by eliminating inhibitory synaptic currents using coapplication of a mixture of the GABA_A_ receptor antagonist gabazine (10 µM) and the glycine receptor antagonist strychnine (1 µM). This synaptic disinhibition did not significantly modulate basal spontaneous AP rate (−11.9 ± 69.5% change from predrug baseline frequency, *n* = 6 cells; Fig. 4*B*), and Epi still increased the spontaneous AP rate in all neurons tested (6/6 cells; Fig. 4*B*). These results demonstrate that adrenergic activation of NAm neurons does not rely solely on either excitatory or inhibitory synaptic transmission.

To further examine whether NAm activation by adrenergic agonists might include a synaptic component, postsynaptic currents of the NAm neurons were recorded during Epi or NE exposures. Most of the isolated spontaneous excitatory (sEPSCs) and inhibitory postsynaptic currents (sIPSCs) were blocked by NBQX (10 µM) and gabazine (10 µM)-strychnine (1 µM) exposure, respectively (Fig. 4, *C* and *D*). Exposure to 10 µM Epi or NE, which modulated AP firing of the NAm neurons, had no effect on the sEPSC or sIPSC frequency (Fig. 4, *E* and *F*). Thus there was no change in spontaneous synaptic transmission induced by adrenergic agonists in a condition where the spontaneous AP firing rate of NAm neurons was increased.

Together, these results suggest that although excitatory synaptic transmission onto NAm neurons can contribute to their spontaneous discharge, it is not critically required for adrenergic activation of NAm neurons, at least in isolated in vitro slices.

#### Modulation of intrinsic membrane excitability by adrenergic agonists.

**Fig. 5.**
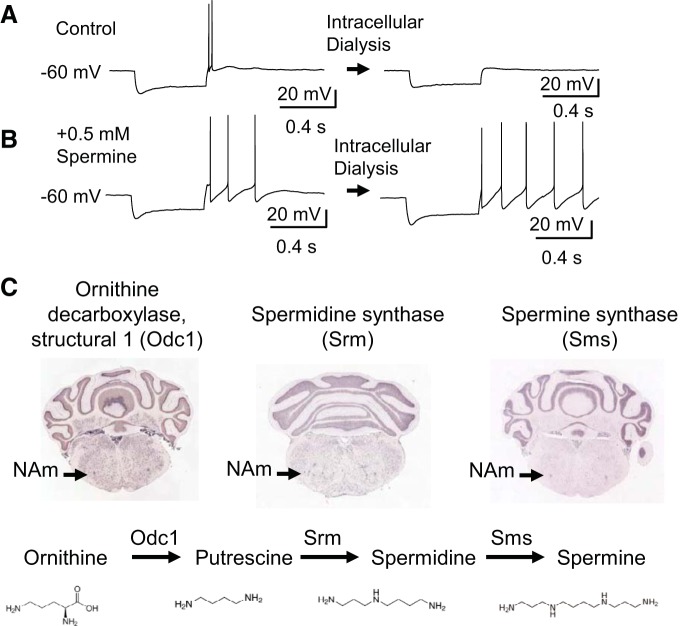
Modulation of intrinsic excitability by adrenergic stimulation. *A* and *B*: intracellular dialysis sensitivity of nucleus ambiguus neurons. Rebound discharge was sensitive to intracellular dialysis and typically disappeared within 5 min after break-in. This run-down effect (*A*) could be reduced by inclusion of 0.5 mM spermine (*B*). *C*: enzymes involved in polyamine biosynthesis (Odc1, Srm, Sms) are expressed in NAm (data from Allen Brain Atlas, www.brain-map.org/).

We next examined whether modulation of intrinsic membrane excitability could be a potential contributor to the adrenergic activation of NAm neurons. We characterized excitability of NAm neurons by whole cell recordings. Similar to previous studies using preparations from mature animals (Johnson and Getting 1991, 1992; Mendelowitz 1996), depolarizing current evoked a train of regular APs interleaved with a large immediate afterhyperpolarization, likely mediated by small-conductance calcium-activated potassium (SK) channels (Lin et al. 2011). A brief hyperpolarizing current typically accompanied by a membrane voltage sag and followed by a rebound AP immediately after termination of hyperpolarizing current was seen in 88% of neurons (88/100 cells; Fig. 5*A*). There was variability in the rebound AP shape during a spike train; one showing an immediate, small afterhyperpolarization (e.g., Fig. 5*A*) can be contrasted with another marked by a larger afterhyperpolarization (e.g., Fig. 5*B*). In both cases, the rebound depolarization was sensitive to intracellular dialysis and became undetectable within ~3 min after membrane break-in (Fig. 5*A*). Previous in situ hybridization analyses showed expression of polyamine biosynthesis enzymes within the NAm (Fig. 5*C*); thus it is possible that wash-out of endogenous polyamine may contribute to the rundown effect. In fact, the rundown could be partly prevented when 0.5–1 mM spermine was supplied in the intracellular solution (Fig. 5*B*), suggesting that endogenous polyamines or related molecules maintain excitability (see discussion). Spermine (0.5 mM) was therefore routinely included in subsequent whole cell current-clamp recordings.

**Fig. 6.**
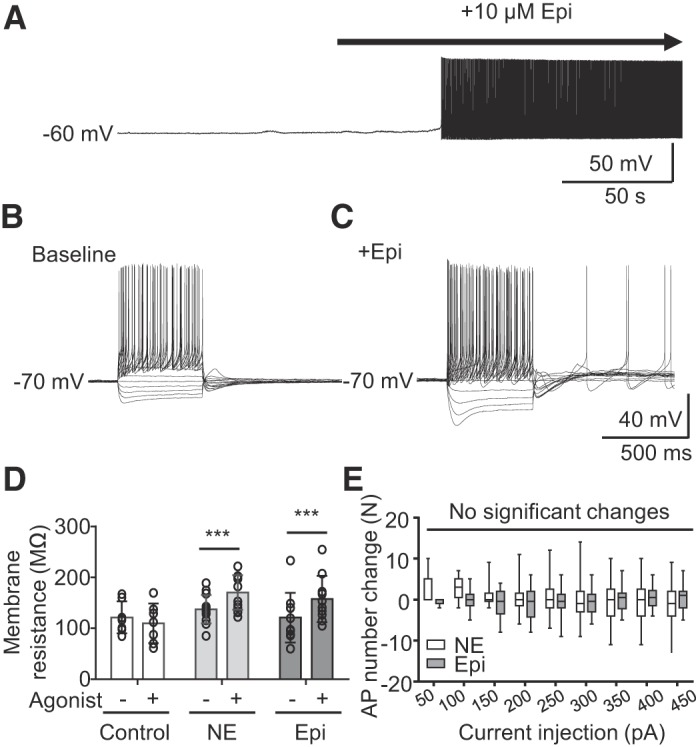
Adrenergic activation of intrinsic membrane excitability of NAm) neurons. *A*. adrenergic activation recorded in a whole cell clamped nucleus ambiguus neuron. Exposure to epinephrine (Epi) led to spontaneous action potential (AP) firing. *B* and *C*: voltage responses to step current injection (−200 pA, +50 pA-increments) in the same neurons at baseline (*B*) and after exposure to Epi (*C*). Note that measurements were made at −60 mV to prevent generation of spontaneous APs. *D*: membrane resistance changes before and after application of each adrenergic agonist. [control, *n* = 7; norepinephrine (NE), *n* = 13; Epi, *n* = 10; repeated-measures ANOVA, *F*(1, 44) = 36.87, *P* < 0.0001). *E*: changes in evoked AP numbers after NE or Epi application. No significant changes were detected [NE, *n* = 13; Epi, *n* = 10; repeated-measures ANOVA, *F*(1, 21) = 0.4821, *P* = 0.495]. ***P < 0.005.

**Fig. 8.**
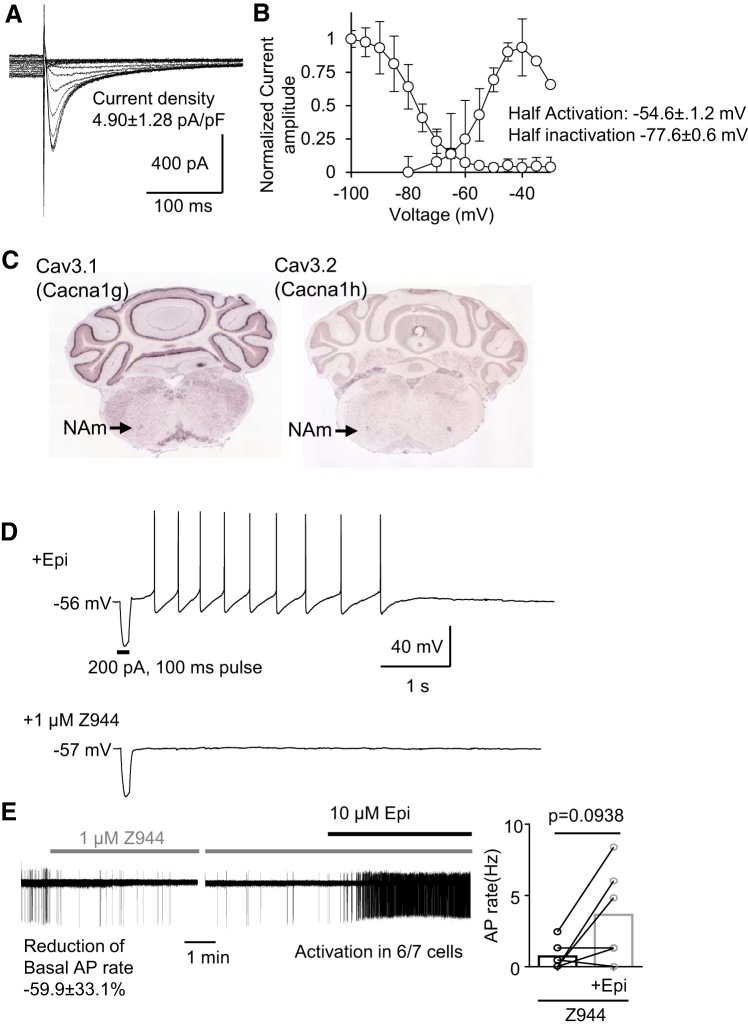
Contribution of T-type calcium current (*I*_CaT_) to the intrinsic membrane excitability of nucleus ambiguus (NAm) neurons. *A*: representative traces of the steady current inactivation of isolated *I*_CaT_ of the NAm neuron. *B*: summary plots of *I*_CaT_ activation/inactivation kinetics. *C*: Cav3.1 (*Cacna1g*) and Cav3.2 (*Cacna1h*) mRNAs are detected in the NAm (data from Allen Brain Institute, www.brain-map.org/). *D*: *I*_CaT_ inhibitor Z944 (1 µM) abolished rebound action potentials (APs) of NAm neurons. *E*: *I*_CaT_ inhibition significantly reduced basal AP firing rate of NAm neurons but did not fully prevent NAm activation by epinephrine (Epi; AP rate increase in 6/7 cells; *P* = 0.094, Wilcoxon matched-pairs signed rank test). Trace shows effect of Z944 on basal AP firing (*left*) and induction of AP by Epi in the same recorded neuron (*right*). Quantitative analysis is shown in bar graph.

Bath application of Epi and NE significantly modified intrinsic excitability of NAm neurons (Fig. 6, *A*–*E*). A representative response is shown in Fig. 5*C*. Consistent with extracellular recordings, Epi exposure triggered continuous spontaneous discharges in whole cell-recorded NAm neurons when held at −60 mV. To avoid spontaneous AP generation, intrinsic membrane excitability was determined at −70 mV. Membrane resistance was measured by applying small hyperpolarizing voltage pulses, to evaluate the amount of leak conductance. As shown in Fig. 6, *B* and *C*, Epi exposure significantly modulated excitability of NAm neurons. Epi and NE increased membrane resistance (Epi: 24.9 ± 11.4%, *n* = 10; NE: 17.7 ± 16.0%, *n* = 13), whereas no changes were seen in control experiments (−15.4 ± 21.6%, *n* = 7; Fig. 6*D*). The increased membrane resistance suggests increased excitability by closure of leak currents. This was accompanied by a larger membrane sag during hyperpolarization mediated by hyperpolarization-activated current (*I*_h_) and enhancement of rebound depolarization, which frequently resulted in AP generation mediated by T-type calcium channels, even at −70 mV (see below). The change in rebound AP was robust, and a single brief hyperpolarization pulse (<500 ms) could evoke recurrent AP firing lasting 3–5 s (see Fig. 8*C*). On the other hand, Epi and NE did not change the number of APs evoked by depolarizing current pulses (Fig. 6*E*). Overall, these adrenergic agonists selectively increased some, but not all, aspects of intrinsic membrane excitability.

#### I_h_ in NAm neurons.

**Fig. 7.**
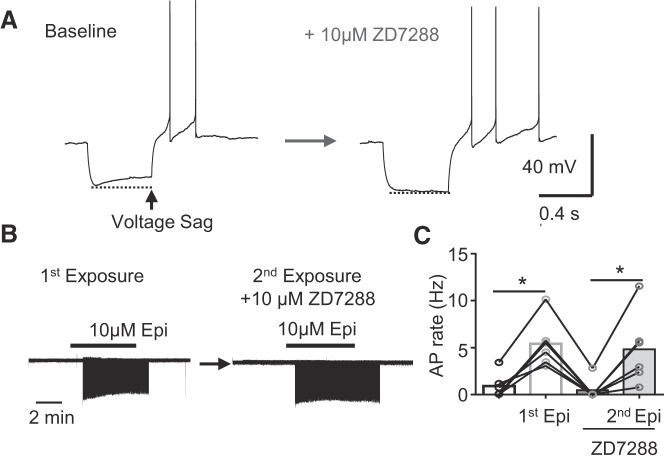
Hyperpolarization-activated current (*I*_h_) is expressed but not required for adrenergic activation of nucleus ambiguus (NAm) neurons. *A*: *I*_h_-mediated membrane sag during hyperpolarizing current was abolished by *I*_h_ inhibitor ZD7288 (10 µM). *Left*, control (baseline); *right*, after ZD7288 treatment. *B* and *C*: ZD7288 at this concentration did not prevent NAm activation by epinephrine (Epi). *B*: representative traces show exposure to Epi (*left*) and second exposure to Epi in the presence of ZD7288 in the same cell (*right*). *C*: quantitative analysis [*n* = 6; repeated-measures ANOVA, *F*(3, 5) = 17.32, *P* = 0.0020]. *P < 0.05.

The voltage sag observed during hyperpolarization (Fig. 7*A*) indicates the presence of *I*_h_, and a previous study in guinea pig suggested that cesium-sensitive putative *I*_h_ (Q-current) is involved in rebound depolarization of NAm neurons (Johnson and Getting 1991). *I*_h_ is required for spontaneous AP generation in various neurons (Resch et al. 2017); however, this was not the case in the NAm. We found that the selective *I*_h_ inhibitor ZD7288 (10 µM) completely abolished the voltage sag (*n* = 3; Fig. 7*A*). However, exposure to ZD7288 had no significant effect on the basal firing rate (4.2 ± 74.0%, *n* = 10), and Epi remained effective at inducing AP firing in all NAm neurons tested [*n* = 6 in repeated experiments (Fig. 7, *B and C*) and in 4 experiments with single Epi exposure (data not shown)]. Thus *I*_h_ is functionally expressed in NAm cholinergic neurons but is not required for adrenergic activation of these cells.

#### T-type calcium current in NAm neurons.

T-type calcium channels are expressed in various pacemaking cells and contribute to spontaneous AP discharges (Chemin et al. 2002; Perez-Reyes 2003). We next examined the potential role of T-type Ca^2+^ current (*I*_CaT_) in NAm excitability. In voltage-clamp recordings, low-voltage-activated current was reliably evoked by a voltage protocol with a mean current density of 4.90 ± 1.28 pA/pF (Fig. 8*A*), a half-activation voltage of −54.63 ± 1.24 mV, and a half-maximum steady stationary inactivation of −77.56 ± 0.63 mV (Fig. 8*B*). The low-voltage-activated current is typically mediated by T-type calcium channels. In fact, the T-type calcium channel genes *Cacna1g* (Cav3.1) and *Cacna1h* (Cav3.2) are likely expressed in the NAm (Fig. 8*C*). The selective *I*_CaT_ inhibitors TTA-P2 (Kraus et al. 2010) and Z944 (Tringham et al. 2012) (1 µM, *n* = 2 for each drug) abolished the inward current as well as the generation of rebound APs in both control solution and following Epi exposure (Fig. 8*D*). The *I*_CaT_ significantly contributes to NAm excitability, because the inhibitor Z944 reduced the spontaneous AP discharge rate by 59.9 ± 33.1% (*n* = 6). However, elimination of *I*_CaT_ by Z944 did not fully prevent NAm activation by Epi [regular spiking (*n* = 4/7) or burst firing (*n* = 2/7); Fig. 8*E*], except in one of the regular-spiking neurons tested (1/7 cells). Therefore, although *I*_CaT_ contributes to basal NAm neuronal excitability, it is not required for Epi-dependent NAm neuronal activation.

#### Persistent sodium current in NAm neurons.

**Fig. 9.**
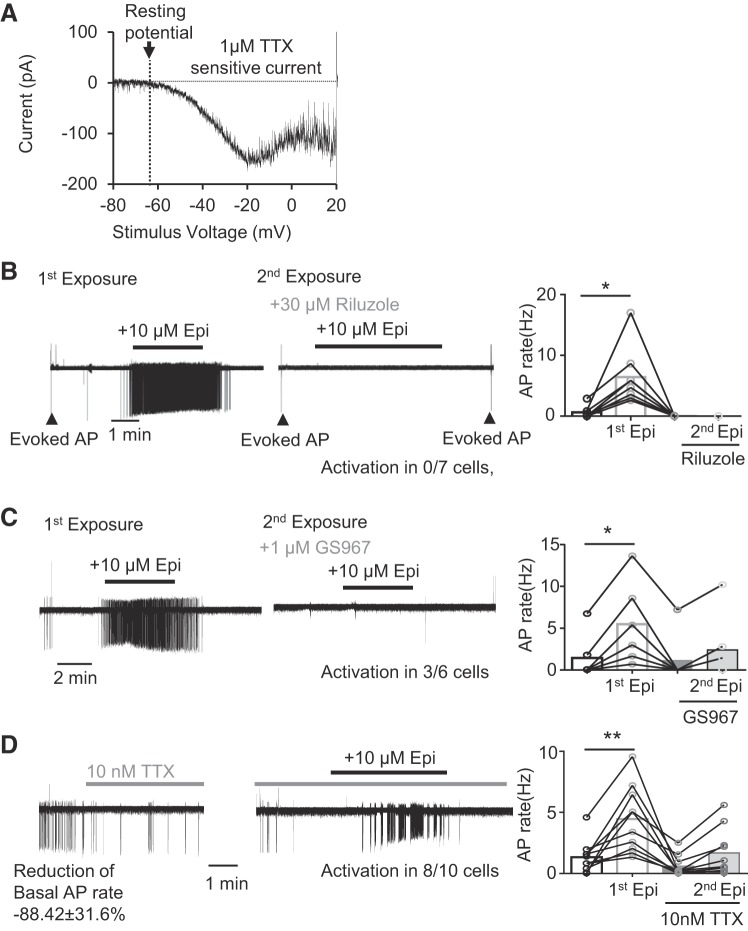
Persistent sodium current (*I*_NaP_) is required for the adrenergic activation of nucleus ambiguus (NAm) neurons. *A*: *I*_NaP_ in a NAm neuron isolated as TTX-sensitive inward current generated during a slow voltage ramp (20 mV/s). *B* and *C*: effect of sodium channel blocker on the action potential (AP) induction by epinephrine (Epi). *B*: nonselective *I*_NaP_ inhibitor riluzole effectively prevented NAm activation by Epi [activation in 0/8 cells; repeated-measures ANOVA, *F*(3, 7) = 14.23, *P* = 0.0057]. Note that riluzole did not prevent APs evoked by electrical stimulation of surrounding tissue (arrowheads). Traces show exposure to Epi (left) and second exposure to Epi in the presence of riluzole in the same cell (*right*). Quantitative analysis is shown in bar graph. *C*: a low concentration of *I*_NaP_-selective inhibitor GS967inhibited activation by Epi in 50% (3/6 cells) of NAm neurons and reduced it in the remaining cells [repeated-measures ANOVA, *F*(3, 5) = 24.76, *P* < 0.0001]. Traces show exposure to Epi (*left*) and second exposure to Epi in the presence of GS967 in the same cell (*right*). Quantitative analysis is shown in bar graph. *D*: a low concentration of TTX (10 nM) reduced spontaneous AP, whereas Epi still increased AP firing rate in the 80% (8/10 cells) of NAm cells tested [repeated-measures ANOVA, *F*(3, 9) = 11.25, *P* = 0.0009]. Representative traces show effect of TTX on basal firing (*left*) and response to Epi in the presence of TTX in the same cell (*right*). Quantitative analysis is shown in bar graph. *P < 0.05; **P < 0.01.

We analyzed the role of *I*_NaP_ in adrenergic activation of NAm neurons. *I*_NaP_ is a slow inward current carried by voltage-gated sodium channels and contributes to spontaneous discharges in various cell types (Bevan and Wilson 1999; Khaliq and Bean 2010, see their discussion; Koizumi and Smith 2008). *I*_NaP_ was isolated as a 1 µM TTX-sensitive component of inward current that developed during a slow voltage ramp (+20 mV/s, 33.6 ± 24.1 pA at −40 mV, *n* = 5; Fig. 9*A*). The isolated *I*_NaP_ emerged near the resting membrane potential of NAm neurons (Fig. 9*A*) and continuously developed to peak at −10~0 mV. The nonselective *I*_NaP_ inhibitor riluzole (30 µM; IC_50_ 2.3~51 µM; Wang et al. 2008; Zona et al. 1998) was highly effective at preventing adrenergic activation of NAm neurons and fully prevented the activation of AP discharges in all 8 NAm neurons tested (Fig. 9*B*). It is notable that even in the presence of riluzole, APs could be evoked by local electrical stimulation, indicating that the observed effect is not due to a nonselective inhibition of sodium current. We further evaluated the contribution of *I*_NaP_ using a specific *I*_NaP_ inhibitor, GS967 (Belardinelli et al. 2013), at the concentration of 1 µM, where the drug has high selectivity for *I*_NaP_ while not fully inhibiting the current in these neurons (Anderson et al. 2014; Belardinelli et al. 2013). *I*_NaP_ inhibition by GS967 fully prevented AP induction in 3/6 cells tested and severely attenuated the response to Epi in the rest of the cells (Fig. 9*C*). These results suggest an important contribution of *I*_NaP_ to the adrenergic activation of NAm neurons.

We next examined the contribution of TTX-insensitive sodium currents to the adrenergic activation. Bath application of 10 nM TTX severely lowered the spontaneous AP rate of NAm neurons (88.4 ± 31.6% reduction from baseline, *n* = 10; Fig. 9*D*), indicating a strong contribution of TTX-sensitive Na^+^ current to basal excitability. However, Epi was still able to increase AP firing rate in 80% of NAm neurons (8/10 cells) tested under this condition (Fig. 9*D*). Thus, although sodium channels that are highly sensitive to TTX are important for basal NAm excitability, TTX-insensitive sodium channels alone are sufficient to mediate Epi-dependent AP increases in NAm neurons.

Finally, we examined the effectiveness of the nonselective sodium current inhibitor flecainide, an antiarrhythmic useful in the control of a variety of supraventricular cardiac tachyarrhythmias, including those bearing *SCN5A* mutations underlying Brugada syndrome (Salvage et al. 2017). Flecainide (20 µM) was partially effective at preventing AP induction and blocked Epi activation in 3/5 cells tested. Thus, in addition to multiple myocardial targets, including Nav1.5 channels in the septal conduction system and cardiac myocytes, this clinically useful peripheral sodium channel blocker may also reduce central vagal nerve activity.

Together, these results suggest that adrenergic activation of NAm neurons requires a persistent sodium current, predominately mediated by sodium channels with low TTX sensitivity.

## DISCUSSION

This study identifies a central adrenergic mechanism regulating the outflow of preganglionic cardiac vagal neurons in the brain stem. We found that two adrenergic agonists, epinephrine and norepinephrine, significantly increase the spontaneous discharge rate of these neurons by acting at α_1_- and β-receptors (Figs. 2 and 3). This adrenergic activation, however, does not require synaptic transmission, because *1*) it occurred even when the postsynaptic receptors were pharmacologically inhibited, and *2*) following agonist exposure, there was no significant change in the frequency of transmitter release at either excitatory and inhibitory synapses onto NAm neurons (Fig. 4). Instead, we observed an increase in their intrinsic membrane excitability leading to vigorous rhythmic discharge (Fig. 6). We also found that mouse NAm neurons express functional *I*_h_, *I*_CaT_, and *I*_NaP_ currents, and of these, the *I*_NaP_ is an important contributor to the intrinsic adrenergic activation, although changes in the other ionic currents are likely involved. These results define a novel mechanism for central adrenergic regulation of parasympathetic output to the heart and define a target excitatory mechanism underlying NAm preganglionic neuron rhythmic activity.

The origin of the adrenergic input remains under study. Because of poor blood-brain barrier permeability of circulating catecholamines (Kostrzewa 2007), brain stem adrenergic neurons located in proximity to NAm (e.g., A1, C1 groups) are a more likely source of synaptic regulation. Optogenetic activation of neurons within the C1 group in the medulla triggers mild bradycardia (Abbott et al. 2013), and the adrenergic mechanism identified in this study may contribute in part to this effect. Given the major role of local brain stem adrenergic neurons in sympathetic cardiac regulation, the adrenergic NAm activation described in this study may be a collateral pathway to counterbalance excessive cardiac sympathetic outflow to limit tachycardia. Alternatively, abnormal coactivation of the cardiac sympathetic and parasympathetic pathways might increase the risk of arrhythmias such as atrial fibrillation (Inoue and Zipes 1987; Ogawa et al. 2007).

Previous studies have demonstrated that adrenergic signaling suppresses inhibitory transmission to cardiac vagal neurons. Thus β-receptor agonists inhibit both sEPSCs and sIPSCs (Bateman et al. 2012), whereas α_1_-agonists (Boychuk et al. 2011) and α_2_-agonists inhibit sIPSCs (Philbin et al. 2010; Sharp et al. 2014). Finally, optogenetic activation of adrenergic neurons within the locus coeruleus inhibits NAm neuronal discharge by selective augmentation of inhibitory currents (Wang et al. 2014). On the basis of these reports, it was unexpected that exposure to adrenergic agonists would have no significant effect on afferent NAm synaptic transmission in our preparation (Fig. 4). The discrepancy could be explained by *1*) the species differences (rat vs. mouse), *2*) the age of animals (neonate/juvenile vs. young adult), and *3*) the precise neuronal population (retrograde tracer labeling vs. ChAT-*cre* reporter) recorded. Among these, age may play a significant role, because rodents show significant postnatal development of the parasympathetic autonomic regulatory system (Kasparov and Paton 1997) during the initial 3 wk of life. In addition, we used thinner brain slices (200 µm) from the larger adult brain stem than previous studies (500–600 µm) using the smaller brain stem of younger animals, where neuronal and synaptic density could be higher, leading to alternative estimates of the contribution of synaptic input.

In addition, our studies reliably detected rebound APs (Fig. 5), which were not reported in studies using preparations from immature animals (Mendelowitz 1996; Mihalevich et al. 1996). Because rebound APs have been observed in adult guinea pig NAm neurons (Johnson and Getting 1991), there could be age-dependent development of this intrinsic membrane excitation mechanism. Alternatively, membrane loading with the lipophilic dye used for cardiac retrograde tracing in previous studies (Mendelowitz 1996; Mihalevich et al. 1996) may have modified excitability of the plasma membrane and masked the intrinsic membrane excitatory mechanism.

A previous study of NAm neurons in adult guinea pig brain stem, using sharp electrode intracellular recording (Johnson and Getting 1991), also identified multiple neuronal populations according to membrane excitability and cell morphology. The pool of preganglionic neurons recorded in this study is consistent with one of the populations displaying delayed rebound action potentials, and the other population may constitute the smaller ChAT-tdTomato^+^ nonpreganglionic neurons and ChAT-tdTomato^−^ neurons (Fig. 1). Our results imply that cardiac preganglionic NAm neurons can be further subdivided on the basis of their response to adrenergic agonists: neurons that show a simple increase in regular spiking and those that generate a burst discharge (Fig. 2). Because of the low chance of encountering the latter, we could not characterize the burst-firing neurons in further detail. Thus it is still unclear whether these two responses may represent two different intrinsic cell populations or a difference attributable to synaptic connectivity. However, because changes in intrinsic membrane excitability were consistently observed in all neurons tested (Fig. 5), it is likely that enhancement of membrane excitability by adrenergic agonists affects both cell types.

Our study suggests that α_1_-receptors are the major mediator of the adrenergic NAm excitability increase, whereas β-receptors may be important for maintenance of basal membrane excitability. The contribution of these receptors to neuronal membrane excitability has been demonstrated in other neurons (Grzelka et al. 2017; Martinez-Peña y Valenzuela et al. 2004; Randle et al. 1986). The α_1_- and β-adrenoceptors are typically coupled with distinct G protein-coupled receptor (GPCR) signaling (i.e., α_1_-receptors with G_q_, β-receptors with G_s_). However, these GPCR pathways cross talk with each other, and β-receptor activation can activate a pathway by direct (Wenzel-Seifert and Seifert 2000; Zhu et al. 1994) or indirect mechanisms (Cervantes et al. 2010; Galaz-Montoya et al. 2017), which may in part explain how distinct adrenoceptors similarly contribute to NAm intrinsic excitability.

Our study also identified multiple ionic currents involved in oscillatory membrane discharges in NAm neurons. We confirmed functional *I*_CaT_ expression in the NAm and its contribution to rebound discharge generation. Consistent with electrophysiological detection, NAm neurons show high expression of Cav3.1 and Cav3.2 mRNA transcripts (Fig. 8*C*; Allen Brain Atlas, www.brain-map.org/). In addition to *I*_CaT_, NAm neurons express a significant *I*_h_, which is involved in membrane oscillation of some cells. Although these currents did not appear to contribute to adrenergic activation in the in vitro preparation, these properties could be involved in complex physiological processes such as vagal outflow during baroreflex and sustained vagal activity in vivo.

Our pharmacological studies (Fig. 9) suggest that *I*_NaP_ is an important component of adrenergic activation of NAm neurons. *I*_NaP_, similarly to the one we identified in this study, contributes to the pacemaking discharge of many neurons (Beurrier et al. 1999; Bevan and Wilson 1999; Khaliq and Bean 2010; Koizumi and Smith 2008; Mercer et al. 2007). *I*_NaP_ is also a mediator of muscarinic agonist-induced spontaneous AP generation of hippocampal CA1 neurons (Yamada-Hanff and Bean 2013), and NAm neurons also possess a similar *I*_NaP_-dependent metabotropic activation mechanism.

Although the specific sodium channels responsible for *I*_NaP_ are not yet determined, our results suggest that TTX-insensitive sodium channels alone are sufficient to mediate adrenergic activation of NAm (Fig. 9). TTX-insensitive channels are encoded by *Scn5a* (Nav1.5), *Scn10a* (Nav1.8), and *Scn11a* (Nav1.9) (Goldin 2001) and are predominantly expressed in cardiac myocytes and peripheral nerves. Brain stem expression of these sodium channels likely contributes to the unique excitation mechanism of NAm and may be fortuitously affected by peripheral sodium channel blockers, such as flecainide.

In summary, our study shows an adrenergic activation mechanism of NAm neurons mediated by modulation of intrinsic membrane excitability that requires *I*_NaP_. The results add an additional site of interaction between the sympathetic and parasympathetic systems within the medulla (DePuy et al. 2013), mediating the finely tuned balance of central autonomic cardiovascular regulation.

## GRANTS

This work was supported by American Heart Association Postdoctoral Fellowship 14POST20130031 (I. Aiba) and National Institute of Neurological Disorders and Stroke Cooperative Agreement U01 NS090340 Center for SUDEP Research (J. L. Noebels).

## DISCLOSURES

No conflicts of interest, financial or otherwise, are declared by the authors.

## AUTHOR CONTRIBUTIONS

I.A. conceived and designed research; I.A. performed experiments; I.A. analyzed data; I.A. interpreted results of experiments; I.A. prepared figures; I.A. drafted manuscript; I.A. and J.N. edited and revised manuscript; I.A. and J.N. approved final version of manuscript.

## References

[B1] Abbott SB, DePuy SD, Nguyen T, Coates MB, Stornetta RL, Guyenet PG Selective optogenetic activation of rostral ventrolateral medullary catecholaminergic neurons produces cardiorespiratory stimulation in conscious mice. J Neurosci 33: 3164–3177, 2013. doi:10.1523/JNEUROSCI.1046-12.2013. 23407970 PMC3596815

[B2] Alboni P, Alboni M, Gianfranchi L Simultaneous occurrence of two independent vagal reflexes: a possible cause of vagal sudden death. Heart 97: 623–625, 2011. doi:10.1136/hrt.2010.221416. 21357371

[B3] Anderson LL, Thompson CH, Hawkins NA, Nath RD, Petersohn AA, Rajamani S, Bush WS, Frankel WN, Vanoye CG, Kearney JA, George AL Jr Antiepileptic activity of preferential inhibitors of persistent sodium current. Epilepsia 55: 1274–1283, 2014. doi:10.1111/epi.12657. 24862204 PMC4126848

[B4] Bateman RJ, Boychuk CR, Philbin KE, Mendelowitz D β Adrenergic receptor modulation of neurotransmission to cardiac vagal neurons in the nucleus ambiguus. Neuroscience 210: 58–66, 2012. doi:10.1016/j.neuroscience.2012.02.033. 22425752 PMC3358477

[B5] Belardinelli L, Liu G, Smith-Maxwell C, Wang WQ, El-Bizri N, Hirakawa R, Karpinski S, Li CH, Hu L, Li XJ, Crumb W, Wu L, Koltun D, Zablocki J, Yao L, Dhalla AK, Rajamani S, Shryock JC A novel, potent, and selective inhibitor of cardiac late sodium current suppresses experimental arrhythmias. J Pharmacol Exp Ther 344: 23–32, 2013. doi:10.1124/jpet.112.198887. 23010360

[B6] Beurrier C, Congar P, Bioulac B, Hammond C Subthalamic nucleus neurons switch from single-spike activity to burst-firing mode. J Neurosci 19: 599–609, 1999. doi:10.1523/JNEUROSCI.19-02-00599.1999. 9880580 PMC6782207

[B7] Bevan MD, Wilson CJ Mechanisms underlying spontaneous oscillation and rhythmic firing in rat subthalamic neurons. J Neurosci 19: 7617–7628, 1999. doi:10.1523/JNEUROSCI.19-17-07617.1999. 10460267 PMC6782508

[B8] Boychuk CR, Bateman RJ, Philbin KE, Mendelowitz D α1-Adrenergic receptors facilitate inhibitory neurotransmission to cardiac vagal neurons in the nucleus ambiguus. Neuroscience 193: 154–161, 2011. doi:10.1016/j.neuroscience.2011.07.024. 21771639 PMC3171606

[B9] Brailoiu GC, Arterburn JB, Oprea TI, Chitravanshi VC, Brailoiu E Bradycardic effects mediated by activation of G protein-coupled estrogen receptor in rat nucleus ambiguus. Exp Physiol 98: 679–691, 2013 a. doi:10.1113/expphysiol.2012.069377. 23104934 PMC3578005

[B10] Brailoiu GC, Benamar K, Arterburn JB, Gao E, Rabinowitz JE, Koch WJ, Brailoiu E Aldosterone increases cardiac vagal tone via G protein-coupled oestrogen receptor activation. J Physiol 591: 4223–4235, 2013 b. doi:10.1113/jphysiol.2013.257204. 23878371 PMC3779113

[B11] Brailoiu GC, Deliu E, Rabinowitz JE, Tilley DG, Koch WJ, Brailoiu E Urotensin II promotes vagal-mediated bradycardia by activating cardiac-projecting parasympathetic neurons of nucleus ambiguus. J Neurochem 129: 628–636, 2014. doi:10.1111/jnc.12679. 24521102 PMC4000260

[B12] Brailoiu GC, Deliu E, Tica AA, Rabinowitz JE, Tilley DG, Benamar K, Koch WJ, Brailoiu E Nesfatin-1 activates cardiac vagal neurons of nucleus ambiguus and elicits bradycardia in conscious rats. J Neurochem 126: 739–748, 2013 c. doi:10.1111/jnc.12355. 23795642 PMC4255462

[B13] Byrum CE, Guyenet PG Afferent and efferent connections of the A5 noradrenergic cell group in the rat. J Comp Neurol 261: 529–542, 1987. doi:10.1002/cne.902610406. 2440916

[B14] Cervantes D, Crosby C, Xiang Y Arrestin orchestrates crosstalk between G protein-coupled receptors to modulate the spatiotemporal activation of ERK MAPK. Circ Res 106: 79–88, 2010. doi:10.1161/CIRCRESAHA.109.198580. 19926878 PMC2818802

[B15] Chemin J, Monteil A, Perez-Reyes E, Bourinet E, Nargeot J, Lory P Specific contribution of human T-type calcium channel isotypes (α_1G_, α_1H_ and α_1I_) to neuronal excitability. J Physiol 540: 3–14, 2002. doi:10.1113/jphysiol.2001.013269. 11927664 PMC2290209

[B16] Colman N, Nahm K, Ganzeboom KS, Shen WK, Reitsma J, Linzer M, Wieling W, Kaufmann H Epidemiology of reflex syncope. Clin Auton Res 14, Suppl 1: 9–17, 2004. doi:10.1007/s10286-004-1003-3. 15480937

[B17] DePuy SD, Stornetta RL, Bochorishvili G, Deisseroth K, Witten I, Coates M, Guyenet PG Glutamatergic neurotransmission between the C1 neurons and the parasympathetic preganglionic neurons of the dorsal motor nucleus of the vagus. J Neurosci 33: 1486–1497, 2013. doi:10.1523/JNEUROSCI.4269-12.2013. 23345223 PMC3727439

[B18] Dergacheva O, Kamendi H, Wang X, Pinol RA, Frank J, Gorini C, Jameson H, Lovett-Barr MR, Mendelowitz D 5-HT_2_ receptors modulate excitatory neurotransmission to cardiac vagal neurons within the nucleus ambiguus evoked during and after hypoxia. Neuroscience 164: 1191–1198, 2009. doi:10.1016/j.neuroscience.2009.09.026. 19772899 PMC2783201

[B19] Dergacheva O, Yamanaka A, Schwartz AR, Polotsky VY, Mendelowitz D Direct projections from hypothalamic orexin neurons to brainstem cardiac vagal neurons. Neuroscience 339: 47–53, 2016. doi:10.1016/j.neuroscience.2016.09.038. 27693474 PMC5118111

[B20] Dyavanapalli J, Byrne P, Mendelowitz D Activation of D2-like dopamine receptors inhibits GABA and glycinergic neurotransmission to pre-motor cardiac vagal neurons in the nucleus ambiguus. Neuroscience 247: 213–226, 2013. doi:10.1016/j.neuroscience.2013.05.039. 23727508 PMC3951879

[B21] Galaz-Montoya M, Wright SJ, Rodriguez GJ, Lichtarge O, Wensel TG β_2_-Adrenergic receptor activation mobilizes intracellular calcium via a non-canonical cAMP-independent signaling pathway. J Biol Chem 292: 9967–9974, 2017. doi:10.1074/jbc.M117.787119. 28442571 PMC5473248

[B22] Goldin AL Resurgence of sodium channel research. Annu Rev Physiol 63: 871–894, 2001. doi:10.1146/annurev.physiol.63.1.871. 11181979

[B23] Grkovic I, Fernandez K, McAllen RM, Anderson CR Misidentification of cardiac vagal pre-ganglionic neurons after injections of retrograde tracer into the pericardial space in the rat. Cell Tissue Res 321: 335–340, 2005. doi:10.1007/s00441-005-1145-1. 15995869

[B24] Grzelka K, Kurowski P, Gawlak M, Szulczyk P Noradrenaline modulates the membrane potential and holding current of medial prefrontal cortex pyramidal neurons via β_1_-adrenergic receptors and HCN channels. Front Cell Neurosci 11: 341, 2017. doi:10.3389/fncel.2017.00341. 29209170 PMC5701640

[B25] Inoue H, Zipes DP Changes in atrial and ventricular refractoriness and in atrioventricular nodal conduction produced by combinations of vagal and sympathetic stimulation that result in a constant spontaneous sinus cycle length. Circ Res 60: 942–951, 1987. doi:10.1161/01.RES.60.6.942. 3594761

[B26] Irnaten M, Wang J, Mendelowitz D Firing properties of identified superior laryngeal neurons in the nucleus ambiguus in the rat. Neurosci Lett 303: 1–4, 2001. doi:10.1016/S0304-3940(01)01693-7. 11297809

[B27] Jameson HS, Pinol RA, Mendelowitz D Purinergic P2X receptors facilitate inhibitory GABAergic and glycinergic neurotransmission to cardiac vagal neurons in the nucleus ambiguus. Brain Res 1224: 53–62, 2008. doi:10.1016/j.brainres.2008.06.012. 18590708 PMC2579798

[B28] Johnson SM, Getting PA Electrophysiological properties of neurons within the nucleus ambiguus of adult guinea pigs. J Neurophysiol 66: 744–761, 1991. doi:10.1152/jn.1991.66.3.744. 1753285

[B29] Johnson SM, Getting PA Excitatory effects of thyrotropin-releasing hormone on neurons within the nucleus ambiguus of adult guinea pigs. Brain Res 590: 1–5, 1992. doi:10.1016/0006-8993(92)91074-O. 1422826

[B30] Kalia M, Fuxe K, Goldstein M Rat medulla oblongata. III. Adrenergic (C1 and C2) neurons, nerve fibers and presumptive terminal processes. J Comp Neurol 233: 333–349, 1985. doi:10.1002/cne.902330304. 2858498

[B31] Kalla M, Herring N, Paterson DJ Cardiac sympatho-vagal balance and ventricular arrhythmia. Auton Neurosci 199: 29–37, 2016. doi:10.1016/j.autneu.2016.08.016. 27590099 PMC5334443

[B32] Kasparov S, Paton JF Changes in baroreceptor vagal reflex performance in the developing rat. Pflugers Arch 434: 438–444, 1997. doi:10.1007/s004240050418. 9211810

[B33] Khaliq ZM, Bean BP Pacemaking in dopaminergic ventral tegmental area neurons: depolarizing drive from background and voltage-dependent sodium conductances. J Neurosci 30: 7401–7413, 2010. doi:10.1523/JNEUROSCI.0143-10.2010. 20505107 PMC2892804

[B34] Koizumi H, Smith JC Persistent Na^+^ and K^+^-dominated leak currents contribute to respiratory rhythm generation in the pre-Bötzinger complex in vitro. J Neurosci 28: 1773–1785, 2008. doi:10.1523/JNEUROSCI.3916-07.2008. 18272697 PMC6671552

[B35] Koizumi K, Kollai M Control of reciprocal and non-reciprocal action of vagal and sympathetic efferents: study of centrally induced reactions. J Auton Nerv Syst 3: 483–501, 1981. doi:10.1016/0165-1838(81)90082-5. 6792258

[B36] Kostrzewa RM The blood-brain barrier for catecholamines – revisited. Neurotox Res 11: 261–271, 2007. doi:10.1007/BF03033571. 17449463

[B37] Kraus RL, Li Y, Gregan Y, Gotter AL, Uebele VN, Fox SV, Doran SM, Barrow JC, Yang ZQ, Reger TS, Koblan KS, Renger JJ In vitro characterization of T-type calcium channel antagonist TTA-A2 and in vivo effects on arousal in mice. J Pharmacol Exp Ther 335: 409–417, 2010. doi:10.1124/jpet.110.171058. 20682849

[B38] Kvetnansky R, Sabban EL, Palkovits M Catecholaminergic systems in stress: structural and molecular genetic approaches. Physiol Rev 89: 535–606, 2009. doi:10.1152/physrev.00042.2006. 19342614

[B39] Lin M, Hatcher JT, Chen QH, Wurster RD, Li L, Cheng ZJ Maternal diabetes increases large conductance Ca^2+^-activated K^+^ outward currents that alter action potential properties but do not contribute to attenuated excitability of parasympathetic cardiac motoneurons in the nucleus ambiguus of neonatal mice. Am J Physiol Regul Integr Comp Physiol 300: R1070–R1078, 2011. doi:10.1152/ajpregu.00470.2010. 21248308 PMC3094040

[B40] Loewy AD, McKellar S, Saper CB Direct projections from the A5 catecholamine cell group to the intermediolateral cell column. Brain Res 174: 309–314, 1979. doi:10.1016/0006-8993(79)90852-7. 487131

[B41] Martinez-Peña y Valenzuela I, Rogers RC, Hermann GE, Travagli RA Norepinephrine effects on identified neurons of the rat dorsal motor nucleus of the vagus. Am J Physiol Gastrointest Liver Physiol 286: G333–G339, 2004. doi:10.1152/ajpgi.00289.2003. 12936913 PMC3062481

[B42] Mendelowitz D Firing properties of identified parasympathetic cardiac neurons in nucleus ambiguus. Am J Physiol 271: H2609–H2614, 1996. 8997322 10.1152/ajpheart.1996.271.6.H2609

[B43] Mercer JN, Chan CS, Tkatch T, Held J, Surmeier DJ Nav1.6 sodium channels are critical to pacemaking and fast spiking in globus pallidus neurons. J Neurosci 27: 13552–13566, 2007. doi:10.1523/JNEUROSCI.3430-07.2007. 18057213 PMC6673100

[B44] Mihalevich M, Neff RA, Mendelowitz D Voltage-gated currents in identified parasympathetic cardiac neurons in the nucleus ambiguus. Brain Res 739: 258–262, 1996. doi:10.1016/S0006-8993(96)00868-2. 8955946

[B45] Ogawa M, Zhou S, Tan AY, Song J, Gholmieh G, Fishbein MC, Luo H, Siegel RJ, Karagueuzian HS, Chen LS, Lin SF, Chen PS Left stellate ganglion and vagal nerve activity and cardiac arrhythmias in ambulatory dogs with pacing-induced congestive heart failure. J Am Coll Cardiol 50: 335–343, 2007. doi:10.1016/j.jacc.2007.03.045. 17659201

[B46] Perez-Reyes E Molecular physiology of low-voltage-activated t-type calcium channels. Physiol Rev 83: 117–161, 2003. doi:10.1152/physrev.00018.2002. 12506128

[B47] Perkins KL Cell-attached voltage-clamp and current-clamp recording and stimulation techniques in brain slices. J Neurosci Methods 154: 1–18, 2006. doi:10.1016/j.jneumeth.2006.02.010. 16554092 PMC2373773

[B48] Philbin KE, Bateman RJ, Mendelowitz D Clonidine, an alpha2-receptor agonist, diminishes GABAergic neurotransmission to cardiac vagal neurons in the nucleus ambiguus. Brain Res 1347: 65–70, 2010. doi:10.1016/j.brainres.2010.06.001. 20553874 PMC2909326

[B49] Randle JC, Bourque CW, Renaud LP α_1_-Adrenergic receptor activation depolarizes rat supraoptic neurosecretory neurons in vitro. Am J Physiol Regul Integr Comp Physiol 251: R569–R574, 1986 10.1152/ajpregu.1986.251.3.R569. 2875661

[B50] Resch JM, Fenselau H, Madara JC, Wu C, Campbell JN, Lyubetskaya A, Dawes BA, Tsai LT, Li MM, Livneh Y, Ke Q, Kang PM, Fejes-Tóth G, Náray-Fejes-Tóth A, Geerling JC, Lowell BB Aldosterone-sensing neurons in the NTS exhibit state-dependent pacemaker activity and drive sodium appetite via synergy with angiotensin II signaling. Neuron 96: 190–206.e7, 2017. doi:10.1016/j.neuron.2017.09.014. 28957668 PMC5637454

[B51] Salvage SC, Chandrasekharan KH, Jeevaratnam K, Dulhunty AF, Thompson AJ, Jackson AP, Huang CL Multiple targets for flecainide action: implications for cardiac arrhythmogenesis. Br J Pharmacol 175: 1260–1278, 2017. doi:10.1111/bph.13807. 28369767 PMC5866987

[B52] Sampaio KN, Mauad H, Michael Spyer K, Ford TW Chronotropic and dromotropic responses to localized glutamate microinjections in the rat nucleus ambiguus. Brain Res 1542: 93–103, 2014. doi:10.1016/j.brainres.2013.10.035. 24177045 PMC3894684

[B53] Sharp DB, Wang X, Mendelowitz D Dexmedetomidine decreases inhibitory but not excitatory neurotransmission to cardiac vagal neurons in the nucleus ambiguus. Brain Res 1574: 1–5, 2014. doi:10.1016/j.brainres.2014.06.010. 24933328 PMC4128000

[B54] Shattock MJ, Tipton MJ ‘Autonomic conflict’: a different way to die during cold water immersion? J Physiol 590: 3219–3230, 2012. doi:10.1113/jphysiol.2012.229864. 22547634 PMC3459038

[B55] Stocker SD, Steinbacher BC Jr, Balaban CD, Yates BJ Connections of the caudal ventrolateral medullary reticular formation in the cat brainstem. Exp Brain Res 116: 270–282, 1997. doi:10.1007/PL00005755. 9348126

[B56] Tringham E, Powell KL, Cain SM, Kuplast K, Mezeyova J, Weerapura M, Eduljee C, Jiang X, Smith P, Morrison JL, Jones NC, Braine E, Rind G, Fee-Maki M, Parker D, Pajouhesh H, Parmar M, O’Brien TJ, Snutch TP T-type calcium channel blockers that attenuate thalamic burst firing and suppress absence seizures. Sci Transl Med 4: 121ra19, 2012. doi:10.1126/scitranslmed.3003120. 22344687

[B57] Wang X, Piñol RA, Byrne P, Mendelowitz D Optogenetic stimulation of locus ceruleus neurons augments inhibitory transmission to parasympathetic cardiac vagal neurons via activation of brainstem α1 and β1 receptors. J Neurosci 34: 6182–6189, 2014. doi:10.1523/JNEUROSCI.5093-13.2014. 24790189 PMC4004807

[B58] Wang YJ, Lin MW, Lin AA, Wu SN Riluzole-induced block of voltage-gated Na^+^ current and activation of BK_Ca_ channels in cultured differentiated human skeletal muscle cells. Life Sci 82: 11–20, 2008. doi:10.1016/j.lfs.2007.10.015. 18068197

[B59] Wenzel-Seifert K, Seifert R Molecular analysis of β_2_-adrenoceptor coupling to G_s_-, G_i_-, and G_q_-proteins. Mol Pharmacol 58: 954–966, 2000. doi:10.1124/mol.58.5.954. 11040042

[B60] Yamada-Hanff J, Bean BP Persistent sodium current drives conditional pacemaking in CA1 pyramidal neurons under muscarinic stimulation. J Neurosci 33: 15011–15021, 2013. doi:10.1523/JNEUROSCI.0577-13.2013. 24048831 PMC3776055

[B61] Yan B, Li L, Harden SW, Epstein PN, Wurster RD, Cheng ZJ Diabetes induces neural degeneration in nucleus ambiguus (NA) and attenuates heart rate control in OVE26 mice. Exp Neurol 220: 34–43, 2009. doi:10.1016/j.expneurol.2009.07.006. 19615367

[B62] Zhu X, Gilbert S, Birnbaumer M, Birnbaumer L Dual signaling potential is common among G_s_-coupled receptors and dependent on receptor density. Mol Pharmacol 46: 460–469, 1994. 7935326

[B63] Zona C, Siniscalchi A, Mercuri NB, Bernardi G Riluzole interacts with voltage-activated sodium and potassium currents in cultured rat cortical neurons. Neuroscience 85: 931–938, 1998. doi:10.1016/S0306-4522(97)00604-0. 9639285

